# Temporal Generalization of Synchronized Saccades Beyond the Trained Range in Monkeys

**DOI:** 10.3389/fpsyg.2018.02172

**Published:** 2018-11-06

**Authors:** Ryuji Takeya, Aniruddh D. Patel, Masaki Tanaka

**Affiliations:** ^1^Department of Physiology, Hokkaido University School of Medicine, Sapporo, Japan; ^2^Department of Psychology, Tufts University, Medford, MA, United States; ^3^Azrieli Program in Brain, Mind & Consciousness, Canadian Institute for Advanced Research, Toronto, ON, Canada

**Keywords:** prediction, synchronization, rhythm, entrainment, learning, eye movement, primate

## Abstract

Synchronized movements with external periodic rhythms, such as dancing to a beat, are commonly observed in daily life. Although it has been well established that some vocal learning species (including parrots and humans) spontaneously develop this ability, it has only recently been shown that monkeys are also capable of predictive and tempo-flexible synchronization to periodic stimuli. In our previous study, monkeys were trained to make predictive saccades for alternately presented visual stimuli at fixed stimulus onset asynchronies (SOAs) to obtain a liquid reward. The monkeys generalized predictive synchronization to novel SOAs in the middle of trained range, suggesting a capacity for tempo-flexible synchronization. However, it is possible that when encountering a novel tempo, the monkeys might sample learned saccade sequences from those for the short and long SOAs so that the mean saccade interval matched the untrained SOA. To eliminate this possibility, in the current study we tested monkeys on novel SOAs outside the trained range. Animals were trained to generate synchronized eye movements for 600 and 900-ms SOAs for a few weeks, and then were tested for longer SOAs. The accuracy and precision of predictive saccades for one untrained SOA (1200 ms) were comparable to those for the trained conditions. On the other hand, the variance of predictive saccade latency and the proportion of reactive saccades increased significantly in the longer SOA conditions (1800 and 2400 ms), indicating that temporal prediction of periodic stimuli was difficult in this range, similar to previous results on synchronized tapping in humans. Our results suggest that monkeys might share similar synchronization mechanisms with humans, which can be subject to physiological examination in future studies.

## Introduction

We have an advanced ability to synchronize our movements with external rhythms, such as dancing and singing to a beat, behaviors seen in every human culture (McNeil, 1997; Savage et al., 2015). Although the brain regions associated with synchronized movements have been elucidated by many neuroimaging and clinical studies (e.g., Chen et al., 2008; Teki et al., 2012; Hove et al., 2013; Lee et al., 2016), direct neuronal recording from behaving animals is crucial for a comprehensive understanding of underlying mechanisms (Merchant et al., 2015; Cadena-Valencia et al., 2018). While some vocal learning species, such as humans and parrots, develop this ability spontaneously (Patel, 2006; Patel et al., 2009; Schachner et al., 2009), it has only recently been shown that macaque monkeys are capable of predictive and tempo-flexible synchronization to periodic stimuli if trained properly (Takeya et al., 2017; Gámez et al., 2018). Sea lions can also acquire this ability with extensive training (Cook et al., 2013; Rouse et al., 2016), and chimpanzees and bonobos spontaneously show some aspects of this ability (Hattori et al., 2013; Large and Gray, 2015). However, unlike monkeys these larger mammals are not suited for extensive neuronal recording and pharmacological manipulations.

In our recent study, monkeys were trained to make eye movements in synchrony with alternately presented silent visual stimuli, and every predictive movement was reinforced by an immediate liquid reward (Takeya et al., 2017). After extensive training on target sequences with stimulus onset asynchronies (SOAs) of 300, 400, 800, and 900 ms, the animals were tested for generalization to 500, 600, and 700-ms SOAs. We found that monkeys were able to generate predictive synchronized saccades with novel tempi, suggesting that they had the capacity for predictive tempo-flexible synchronization.

However, one might argue for another possibility. In our previous experiments, the animals might learn sequential saccades with specific intervals during training, rather than genuinely showing flexible entrainment. For example, when monkeys were presented with a novel 600-ms SOA, they might sample saccade sequences produced for 400 and 800 ms intervals so that their mean inter-saccadic interval (ISI) became roughly 600 ms. A similar strategy could be used for temporal generalization of synchronized tapping in monkeys which were also examined only within the trained range (Gámez et al., 2018).

Thus, an important question is whether true tempo flexibility exists in monkey synchronization to a metronome. Humans easily show generalization in many perceptual and motor tasks, including generalization outside the trained range. Other species do not always exhibit this flexibility. For example, European starlings trained to discriminate between rising and falling tone sequences do not generalize this discrimination when the sequences are frequency-shifted (“transposed”) beyond the trained range (Hulse and Cynx, 1985). It has also been demonstrated that the generalization within the trained range is imperfect but is much better than that beyond the trained range when pigeons are tested for color discrimination (Thomas and Williams, 1963). In contrast, monkeys may have an ability for out-of-range generalization; in one study, rhesus monkeys trained on a luminance discrimination task were able to choose a brighter stimulus from a pair with various luminances, including values outside the trained range (Flagg et al., 1974).

To address the issue, we examined whether monkeys could generalize synchronized movements beyond the trained range. Specifically, the animals were trained for synchronized eye movements for certain SOAs and then were tested for novel SOAs longer than the trained ones. Because a saccade sequence with a longer interval cannot be generated by any combination of saccade sequences for trained SOAs, true generalization could be examined. Furthermore, we also explored the upper limit of synchronization in monkeys by extending the interval. In humans, synchronized tapping has been shown to become increasingly difficult for SOAs longer than 1800 ms, with reactive (rather than predictive) taps sharply increasing in frequency (Mates et al., 1994; Engström et al., 1996; Miyake et al., 2004; Repp, 2006; Tokushige et al., 2018). We show that monkeys exhibit out-of-range synchronization and also have a temporal limit of synchronization similar to humans.

## Materials and Methods

### Animal Preparation

Two male and one female Japanese monkeys (*Macaca fuscata*, 6–9 kg, 6–8 years old, monkeys I, J, and K, respectively) were used. Two of them (J and K) also participated in our previous study (Takeya et al., 2017). All experimental protocols were evaluated and approved in advance by the Animal Care and Use Committee of Hokkaido University. The procedures for animal preparation are described in detail elsewhere (Tanaka, 2005). Briefly, a head holding device and scleral search coil were implanted in separate surgeries under general isoflurane anesthesia. Analgesics were administered during and a few days following each surgery. Behavioral training and experiments were undertaken after full recovery from the surgery.

### Experimental Procedures

The experimental setup and the basic configuration of behavioral paradigms were identical to the previous study (Takeya et al., 2017), although in this study we modified the range of stimulus intervals and some other parameters such as the trial length and the amount of reward (see below). During the training and experimental sessions, the monkey’s head was secured to the primate chair and the horizontal and vertical eye position were continuously recorded using the search coil technique (MEL-25, Enzanshi Kogyo). Experiments were controlled by a Windows-based real-time stimulus presentation and data acquisition system (TEMPO, Reflective Computing) that updated stimulus events at 200 Hz and acquired the eye movement data at 1 kHz.

**FIGURE 1:**
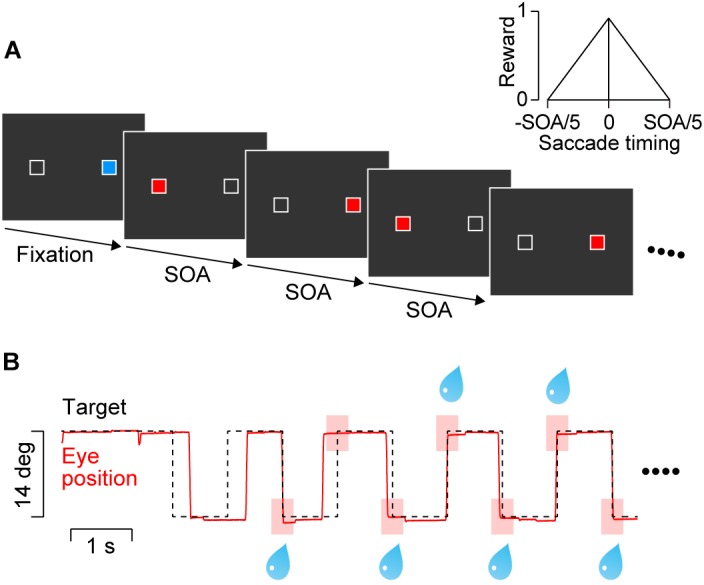
Behavioral paradigm. **(A)** Two white landmarks (1° squares) were presented horizontally (14° apart) throughout the trial. A blue square within either landmark served as the initial fixation target. After a random fixation period, a saccade target (red square) was presented at the opposite landmark location. The target alternated with a constant stimulus onset asynchrony (SOA) that was randomly chosen from 600 to 2400 ms in each trial. Only 600 and 900-ms SOAs were presented during the training sessions for two monkeys. **(B)** Data from a representative trial with a 900-ms SOA. The animal obtained a liquid reward for every predictive saccade that arrived at the target location (<3°) within ± 20% SOA from target onset (pink rectangles). The amount of reward was proportional to the inverse of time difference between saccade termination and target onset (inset in panel **A**).

Visual stimuli were presented on a 27-inch liquid crystal display (XL2720Z, BenQ, refresh rate: 144 Hz) that was positioned 40 cm from the eyes and subtended 73° × 46° of visual angle. Throughout the experiment, two landmarks (1° white square contours) were presented ± 7° horizontally (Figure 1A) on the empty screen. The blue initial fixation target (33.9 cd/m^2^) and red saccade target (33.9 cd/m^2^) were presented within the landmark. Each trial started when animals achieved initial fixation for 1750–2250 ms. Then, the blue target was extinguished and the red saccade target appeared on the other landmark location. The saccade target was alternately presented at the landmark locations with a constant SOA of 600, 900, 1200, 1800, or 2400 ms. As described below, only target sequences with 600 and 900-ms SOAs were presented during the training sessions in two monkeys. Once the initial fixation target disappeared, the stimulus sequence lasted for 14400–15000 ms, which contained 6–25 target steps. The target was visible until the next target presentation on the opposite side of the screen. Because the initial fixation period and the SOA varied from trial to trial, monkeys were unable to predict the timing of the initial two stimuli in the sequence. The trial was aborted immediately if either the first or second saccade had a latency shorter than 150 ms, or if the eyes deviated 3.5° vertically from the horizontal meridian.

The animals were trained to synchronize their eye movements with target onset. They received an immediate reward for each saccade that terminated at the target location (<3°) within ± 20% SOA from the onset of the fourth or later target in the sequence (as in Takeya et al., 2017, Figure 1B, pink rectangles). The maximal amount of reward for each saccade was adjusted by changing the open interval of solenoid valve so that the total amount of reward in each trial was roughly the same across SOAs (i.e., 0.14, 0.22, 0.29, 0.43, and 0.58 mL for the 600, 900, 1200, 1800, and 2400-ms SOA conditions, respectively). In addition (and differently from Takeya et al., 2017), to facilitate accurate synchronization the amount of reward was scaled depending on the time difference between saccade end and target onset (Figure 1A, inset). To prevent animals from generating multiple saccades for a single target presentation (to earn more reward), no reward was delivered for saccades with short ISIs (<33% SOA).

To test whether the animals could generalize synchronized movements for novel SOAs beyond the trained range, the two male monkeys (I and J) were trained on 600 and 900-ms SOAs. Then, these animals performed three test sessions consisting of trials with SOAs of 600, 900, 1200, 1800, and 2400 ms. The remaining female monkey (K) was extensively trained for all SOA conditions.

### Data Acquisition and Analysis

**FIGURE 2:**
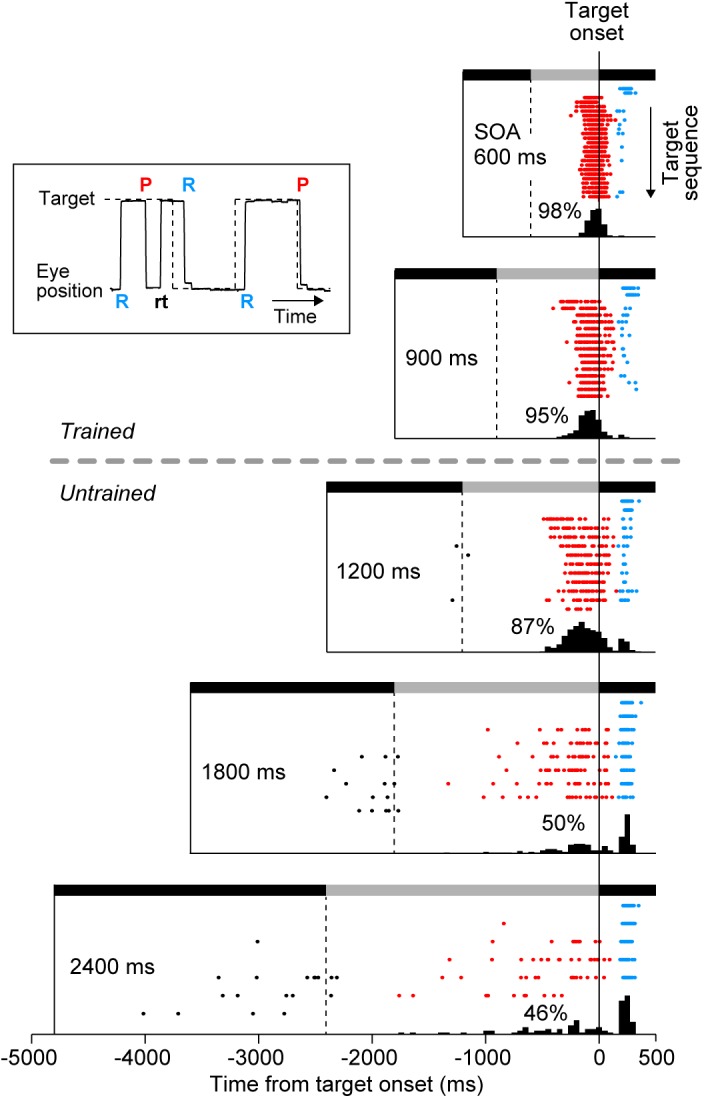
Distribution of saccade latency in a representative experiment. Inset plots a sample record of eye and target position, and shows the definition of three types of saccades. “P” and “R” indicate the onsets of predictive and reactive saccades, respectively. Reactive saccades were generated within 150–500 ms following the target onset. Predictive saccades were directed away from the current target and toward the upcoming target, and were sometimes followed by return saccades (“rt,” black). Predictive saccades were also directed toward the target when generated within 150 ms following the target onset. Rasters plot saccade timing in the direction of the target (target onset is at time zero). Each row of the raster plots corresponds to the target sequence in many trials, with the first target in the trial shown at the top of raster lines. Note that saccades for the initial two targets were always reactive (blue dots) because the SOA varied from trial to trial. Black and gray horizontal bars indicate target locations that alternated at the SOA. Histograms below the rasters indicate the distribution of saccade latency for the third and subsequent targets. Numbers indicate the proportions of predictive saccades computed for the third and later saccades in the sequence.

Eye movement data were digitized and sampled at 1 kHz, and were saved in files along with event timestamps during experiments. Data were analyzed offline using MATLAB (MathWorks). Saccade latency was measured as the time of saccade initiation relative to target onset, while during experiments we monitored times of saccade termination to control the amount of reward (Figure 1). Typically, the duration of a saccade in our experimental condition was approximately 40 ms. For quantitative analysis, we defined (1) predictive, (2) reactive, and (3) return saccades (Figure 2, inset). Predictive saccades were those directed away from the current target (or those generated within 150 ms of target onset, red “P” in Figure 2 inset). Reactive saccades were those generated within 150–500 ms following the target onset (blue “R”), because reactive saccade latencies were generally longer than 150 ms in our experimental condition (Takeya et al., 2017). Return saccades were those directed toward the visible target, usually following the early predictive saccades in the opposite direction (black “rt”). For quantification, we computed the proportion of reactive saccades, the normalized variance of predictive saccade latency (SD of saccade latency divided by SOA), and the SD of reactive saccade latency in each experimental session. For each animal, the data were collected from three daily sessions. On average, each session contained 155 ± 55 trials (SD, *n* = 9, ranged from 78 to 258 trials), which corresponded 2462 ± 802 saccades. A one-way analysis of variance (ANOVA) for the means of different conditions and the subsequent *post hoc* multiple comparisons (*t*-test with Bonferroni correction) were conducted to evaluate the statistical difference of each parameter. These statistical tests were performed on both individual sessions (*n* = 9) and the averaged data for each monkey (*n* = 3) comparing across conditions.

## Results

We examined whether monkeys were capable of generating synchronized movements for SOAs outside of the trained range. After initial training on 600 and 900-ms SOAs, two monkeys (I and J) performed 3 test sessions consisting of trials with 600, 900, 1200, 1800, and 2400-ms SOAs. Figure 2 illustrates the data from a single experiment in monkey I. For each SOA condition, the timing of saccades are shown by rasters, and the associated latency histograms are constructed from the third and later saccades in the sequence. Since the SOA was randomly chosen in each trial, saccades to the first and second targets (upper two rows of raster lines) were reactive and had latencies longer than 150 ms (blue dots) in all SOA conditions. In contrast, most saccades for the third and later target were predictive (red), especially for short SOAs. The distribution of saccade latencies centered at negative value, which was similar to the “negative asynchrony” known in synchronized tapping in humans (Engström et al., 1996; Mates et al., 1994; Miyake et al., 2004). The mean saccade latencies for the third and the later targets were significantly shorter than those for the first and second targets in all SOA conditions (*t*-tests with Bonferroni correction, *t*_548_ = 18.1, *t*_379_ = 12.8, *t*_285_ = 14.8, *t*_203_ = 11.7, *t*_192_ = 7.5 for 600, 900, 1200, 1800, and 2400-ms SOAs, respectively, *p*s < 0.001). However, the proportion of reactive saccades gradually increased for longer SOA conditions (2, 5, 12, 45, and 47%), suggesting that the animal had a difficulty in predicting the target timing when SOA was long. Although the animal obtained a small amount of reward for reactive saccades in the condition with longer SOAs (because reactive saccades could occur within ± 20% SOA), a significant number of predictive saccades were still observed, indicating that the animal attempted to generate synchronized saccades.

**FIGURE 3:**
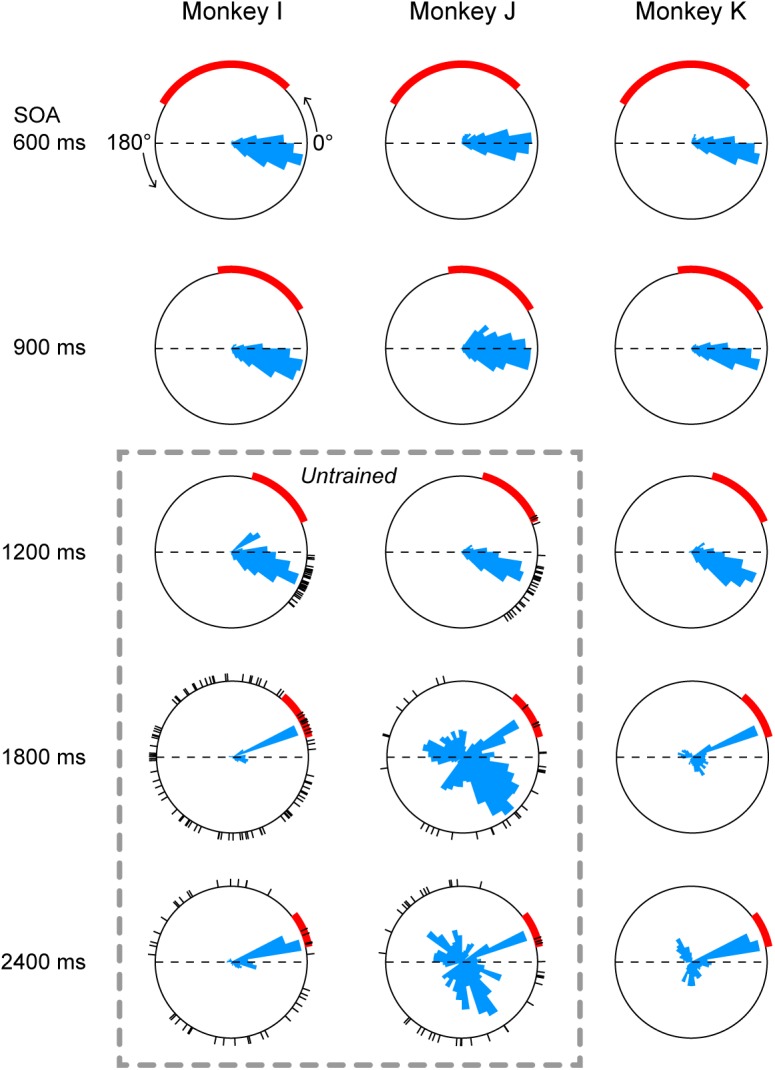
Circular histograms of saccade timing in all conditions. Each panel plots the data obtained from three daily sessions. 0° indicates target onset in the direction of saccade, while 180° indicates target onset in the opposite direction (thus the arc between 0° and 180° represents one SOA). Red arcs indicate the range of reactive saccade latency (150–500 ms after target onset). The circular histogram on each panel is normalized for its peak value. Dashed rectangle indicates untrained conditions. Black short lines on the circles indicate saccade timing relative to the target onset in the first three trials during the very first session of the untrained sequence.

Histograms below rasters in Figure 2 also show that the distributions of saccade latency differed across SOA conditions, possibly because the accuracy of temporal prediction depended on the SOA. To directly compare the distributions of saccade timing between conditions, Figure 3 plots circular histograms of saccade latency for the third and later target for all SOA conditions. In all three monkeys, the circular distributions of saccade latency were similar between the 900 and 1200-ms SOA conditions, while the phase of predictive saccades slightly advanced (i.e., saccades become more predictive) in the 1200-ms SOA condition. In monkey I, reactive saccades clearly increased in the 1800 and 2400-ms SOA conditions (red arc indicates the range of reactive saccades). Monkey J generated many predictive saccades with very variable latency in the two longest SOA conditions, but also frequently made reactive saccades. Monkey K was extensively trained for all SOAs (Methods), but many saccades were reactive in the 1800 and 2400-ms SOA conditions.

To assess the variance of saccade latency for different SOAs, each saccade was converted into a vector having unity length and an angle corresponding to the phase in the circular distribution. Then, we computed the sum of vectors in each SOA condition. For example, the vector length was 0.95, 0.93, 0.90, 0.86, and 0.84 for the five panels of monkey I in Figure 3. The vector lengths computed for all experimental sessions (3 monkeys × 3 sessions each) averaged 0.91 ± 0.03 (SD), 0.92 ± 0.02, 0.92 ± 0.02, 0.79 ± 0.12, and 0.75 ± 0.12 for 600, 900, 1200, 1800, and 2400-ms SOAs, respectively. A one-way ANOVA and *post hoc* multiple comparisons detected significant difference between the three shortest (600, 900, and 1200-ms) and the two longest (1800 and 2400-ms) SOA conditions (ANOVA: *F*_4,40_ = 10.2, *p* < 0.0001, *t*-test with Bonferroni correction: *t*_16_ = 3.7, 4.8, 3.9, 5.0, 3.7, and 4.8, *p*s < 0.05) but not within the groups (*t*_16_ = 0.9, 0.1, 0.7, and 0.9, *p*s > 0.99). Thus, prediction accuracy of next stimulus timing in the 1200-ms SOA condition was comparable to those in the 600 and 900-ms SOA conditions, while temporal prediction in the 1800 and 2400-ms conditions was relatively inaccurate. When the data obtained from three sessions were averaged for each monkey, a one-way ANOVA again detected significant SOA effect (*F*_4,10_ = 4.3, *p* < 0.05), while *post hoc* multiple comparisons failed to find significant difference between any pair of SOAs (because of small number of animals).

**FIGURE 4:**
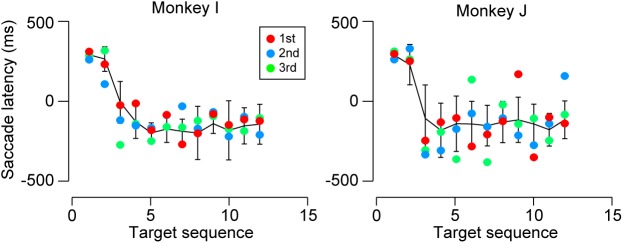
Saccade latencies for 1200-ms SOA during the first test session. Colored dots indicate the data for the initial three trials. Black lines connect the means of subsequent saccade latencies as a function of target sequence location in the same session. Error bars denote ± SD. Note that both monkeys generated predictive saccades for the novel stimulus sequence during the very first trials.

Predictive saccades were observed even during the very first trials when the animals were exposed to a novel sequence of 1200-ms SOA. Black tick marks in Figure 3 (untrained conditions) plot the timing of the third and later saccades in the sequence during the initial three trials in the first experimental session. Both monkeys I and J consistently generated predictive saccades for 1200-ms SOA, while saccade timing relative to target appearance greatly varied for 1800 and 2400-ms SOAs. Figure 4 illustrates the time courses of saccade latency for the sequence of 1200-ms SOA, comparing the data for the initial three trials (colored dots) with the means (± SDs) for the subsequent trials (black lines). The data clearly show that both animals generated predictive saccades and were able to synchronize with the novel target sequence after a few cycles even during the very first trials.

**FIGURE 5:**
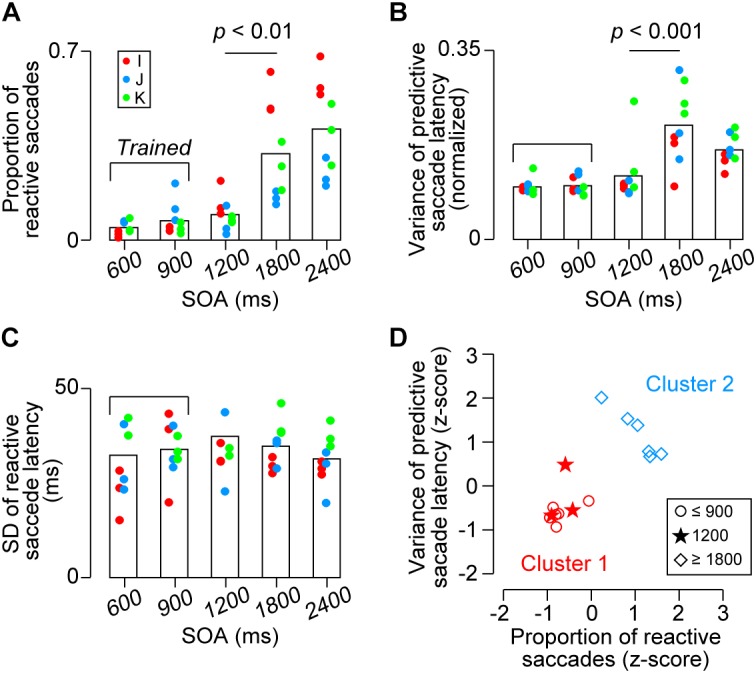
Quantitative data. **(A)** Proportion of reactive saccades. Bracket indicates trained SOAs for monkeys I and J (600 and 900 ms). **(B)** Normalized variance of predictive saccade latency (SD divided by SOA). **(C)** Variance of reactive saccade latencies. Note that the values in *A* and *B* were significantly greater for the 1800 and 2400 ms conditions than the other conditions (*p*s < 0.01), while no significant difference was found across conditions in **(C)** (*p*s > 0.99). **(D)** Classification of the data shown in **(A,B)** for each monkey. Different shapes of symbols indicate SOA conditions. Colors indicate the results of k-means clustering and show that the behavioral performance for the short SOAs (600 and 900 ms, trained sequence, red circles) differed from that for the long SOAs (1800 and 2400 ms, untrained, blue diamonds). Note that the data for the novel 1200-ms sequence were grouped into the trained condition (red stars).

To further examine the difference between the SOA conditions, we also computed several other parameters for each experiment. Figure 5A compares the proportions of reactive saccades for the third and later targets in different SOA conditions. A one-way ANOVA for the data of 9 sessions (3 sessions for each monkey) detected significant effects of SOA (*F*_4,40_ = 18.3, *p* < 10^-8^) and *post hoc* multiple comparisons for each pair of SOA conditions showed significant difference between the 1200-ms and the longer SOA conditions (*t*_16_ = 3.6 and 5.3, *p*s < 0.05). No significant difference was found within the three shortest SOA conditions and the two longest SOA conditions (*t*_16_ = 1.2, 2.3, 0.6 and 1.1, *p*s > 0.36). Thus, the proportion of reactive saccades for 1200-ms SOA was as small as those for 600 and 900-ms SOAs, indicating that the animals could generalize predictive synchronized movements for target sequence beyond the trained range.

One might argue that the increased proportion of reactive saccades in the longer SOA conditions could be attributed to the fact that the variation of temporal prediction was proportional to the SOA (i.e., scalar property) and a delayed prediction would result in a reactive response. In this case, the normalized variation of predictive saccade latency calculated from the actual data in the longer SOA conditions would become less than those in the other conditions because the longer tail of predictive saccade distribution (i.e., >150 ms) had been omitted. To test this, Figure 5B compares the normalized variation of predictive saccade latency (SD of predictive saccade latency divided by SOA) across conditions. The values differed across SOAs (*F*_4,40_ = 12.4, *p* < 10^-5^), and the *post hoc* tests for each pair of SOA conditions showed that the value for 1800-ms SOA was significantly greater than those for the three shortest SOAs (*t*_16_ = 4.8, 4.8, and 3.4, *p*s < 0.05), while the values were not different between any pair of the three shortest SOAs (600, 900, and 1200 ms) or between the two longest SOA conditions (*t*_16_ = 0.1, 1.0, 1.0, and 2.1, *p*s > 0.53). Thus, the data show that the precision of predictive saccades was comparable between 600, 900, and 1200-ms SOAs, while it became greater in the remaining conditions. These results suggest that temporal prediction was difficult for the l800 and 2400-ms SOAs, and that the increased proportion of reactive saccades for longer intervals could not solely attributed to the greater variation of temporal prediction. In contrast, when we compared the variance of reactive saccades for different SOAs, there was no difference across conditions (Figure 5C, *F*_4,40_ = 0.62, *p* = 0.64).

We also examined the effects of SOAs for the data averaged for each animal. The proportions of reactive saccades averaged 5 ± 3% (SD, *n* = 3), 7 ± 5%, 10 ± 5%, 33 ± 20%, and 42 ± 18%, for 600, 900, 1200, 1800, and 2400-ms SOAs, respectively, and were statistically different (one-way ANOVA, *F*_4,10_ = 5.63, *p* < 0.05). The normalized variance of predictive saccade latency also differed significantly depending on the SOA (*F*_4,10_ = 5.88, *p* < 0.05). For both measures, *post hoc* multiple comparisons failed to detect significant difference between any pair of SOA conditions, because the number of subjects was only three. Therefore, we asked whether the data for 1200-ms SOA were comparable to either the shorter (trained) or longer (untrained) SOA conditions using the k-means clustering procedure. Figure 5D shows that the data for 1200-ms SOA (stars) were classified into the group of the shorter SOA conditions (red symbols), indicating that the behavioral performance for the novel 1200-ms sequence was indistinguishable from that for the trained SOAs. Taken together, our results showed that all parameters of saccade latency for 1200-ms SOA were comparable to those for the trained SOAs, whereas saccades in the 1800 and 2400-ms SOA conditions tended to be reactive and variable suggesting that temporal prediction of next stimulus was difficult in this range.

## Discussion

To examine how flexibly monkeys could generalize predictive synchronized movements to metronomic stimuli at various tempi, two Japanese monkeys were extensively trained to make eye movements in synchrony with alternately presented visual stimuli of 600 and 900-ms SOAs, and then were presented with longer SOAs (1200, 1800, and 2400 ms) along with the trained intervals. We found that the accuracy and precision of predictive saccades for 1200-ms SOA were comparable to those for the trained intervals (Figure 3), indicating that monkeys were able to generalize synchronized movements beyond the trained range. On the other hand, the proportion of reactive saccades significantly increased for 1800 and 2400-ms SOAs (Figure 5A), even in the third monkey trained for all SOAs, suggesting that there might be a temporal limit of predictive synchronization.

**FIGURE 6:**
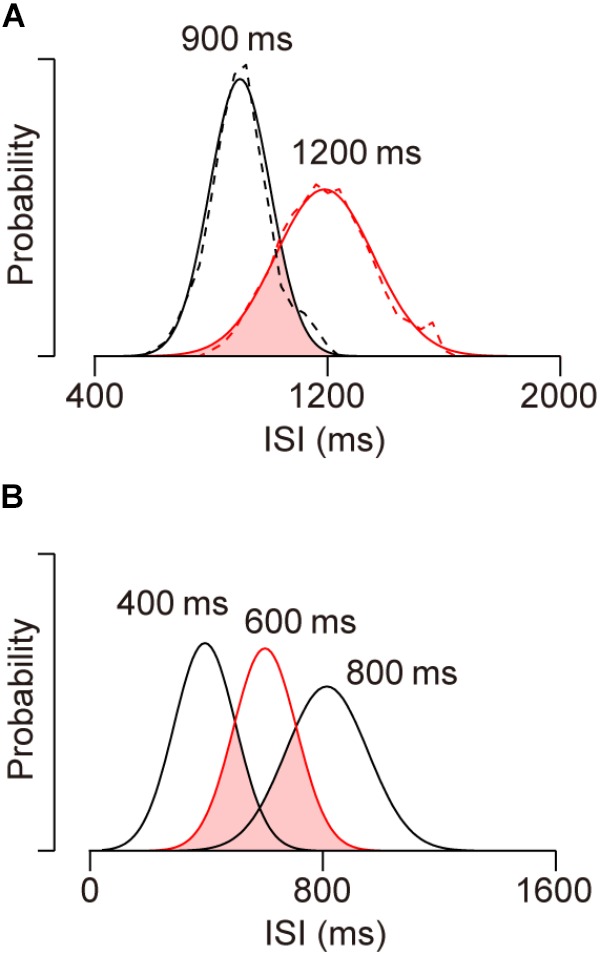
Distributions of inter-saccadic intervals (ISIs) of predictive saccades in different conditions. **(A)** Dashed traces show data from two monkeys (I and J) for 900 (black, trained) and 1200-ms (red, untrained) SOA conditions. Solid traces indicate normal distributions with the same means and SDs as actual data. Note the limited distribution overlap (pink), indicating that the ISI distribution for novel 1200-ms SOA cannot be accounted for by sampling from learned saccade sequence for 900-ms SOA. **(B)** Data obtained in the previous study (Takeya et al., 2017) for SOAs of 400 (trained), 600 (red, untrained) and 800 (trained) ms. Approximately 69% of the ISI distribution for the novel 600-ms SOA condition overlaps with the learned 400 and 800-ms SOA distributions.

As noted in the Section “Introduction,” synchronized movements beyond the trained range cannot be generated by making sequential saccades with learned ISIs. To quantify how unlikely this is, we attempted to reproduce the distribution of ISIs for 1200-ms SOA by randomly resampling from saccade timing data for 900-ms SOA. Dashed traces in Figure 6A show the distributions of ISIs for the sequence of predictive saccades in the 900 and 1200-ms SOA conditions for two monkeys (I and J). For the subsequent analyses, we substituted the traces with Gaussian curves with mean and SD that were derived from the actual data (continuous traces). The distributions for the two conditions overlap by only 36% (pink area), and it seems to be impossible to reproduce the greater ISIs for 1200-ms SOA from the distribution in the 900-ms SOA condition. To test this, we conducted a simulation by randomly taking 1000 samples from the intersected region and calculated the mean ISI. After 1000 repeats of this procedure, the mean ISIs averaged 1011 ± 3 ms (SD), which was 181 ms shorter than the mean of actual ISI for 1200-ms SOA (1192 ms). The probability of the mean of resampled data reaching the actual value was vanishingly small (*p* < 10^-734^). Thus, the predictive saccades for 1200-ms SOA cannot be explained by the distribution of learned ISIs for 900-ms SOA, indicating that the animals were able to apply a synchronization strategy to novel tempi beyond the trained range.

We also conducted the same analysis to estimate how likely generalization *within* the trained range would be if the subjects generated saccade sequence of learned ISIs. In our previous study, monkeys were initially trained with 300, 400, 800, and 900-ms SOAs, and then were tested generalization for 500, 600, and 700-ms SOAs, which were all within the trained range (Takeya et al., 2017). In the simulation, we tried to reproduce the ISI distribution for a 600-ms SOA by randomly resampling the data for 400 and 800-ms SOAs. In Figure 6B, the three Gaussian curves indicate ISI distributions for 400, 600, and 800-ms SOAs with the means and SDs taken from the actual data (Takeya et al., 2017, monkeys J and X). The area of interception (pink) was 69% of the ISI distribution for 600-ms SOA, suggesting that the generalization in the 600-ms SOA condition could be explained, at least partly, by the combination of learned saccade sequence with trained ISIs. To reproduce the ISI distribution for 600-ms SOA, 500 data points were randomly resampled from each of the overlapped distributions (i.e., from the ISI sequence for 400 and 800-ms SOAs). After 1000 repeats, the means of the resampled distributions averaged 618 ± 3 ms, which was slightly greater than the actual data (607 ms, *p* < 10^-5^). Importantly, the SDs of the resampled distributions averaged 128 ± 2 ms and was also greater than the actual value (113 ms, *p* < 10^-8^). When the variation of ISI was assessed by calculating the coefficient of variation, the value for the resampled data (0.205 ± 0.004) was greater than that for the actual data (0.186, *p* < 10^-6^), likely because the overlapped distribution for the two SOA conditions was wide and bimodal (Figure 6B, pink area).

When we performed the same analysis by resampling data from the whole distributions for 400 and 800-ms SOAs, the means of resampled distributions averaged 609 ± 4 and were comparable to the actual data for 600-ms SOA (*p* = 0.23), while the SDs of resampled data were again significantly greater than the actual value (234 ± 3 ms, *p* < 10^-277^). These results indicate that the ISI distribution for 600-ms SOA could not be produced by generating learned saccade sequences for 400 and 800-ms SOAs. Taken together with the results of out of range generalization, our data demonstrated that monkeys were able to immediately acquire a new target sequence and generate tempo-flexible synchronized movements.

On the other hand, the properties of sequential saccades for 1800 and 2400-ms SOAs were qualitatively different from those in the 1200-ms SOA condition. Both the increased proportion of reactive saccades (Figure 5A) and the greater variation in timing of predictive saccades (Figure 5B) for the 1800 than 1200-ms SOAs indicated that monkeys were unable to accurately entrain to target sequence in this range. Like our monkeys, it has been shown in humans that precise, predictive synchronized tapping is difficult for stimulus sequences with 1800-ms or longer SOAs and is nearly impossible for sequences with 3000-ms SOA (Mates et al., 1994; Engström et al., 1996; Miyake et al., 2004; Repp, 2006; Tokushige et al., 2018, but see Repp and Doggett, 2007). In humans, the upper limit of synchronization is relevant to the temporal limit of beat perception, and is thought to be determined by the capacity of temporal integration of working memory (Pöppel, 1997). The existence of an upper limit of synchronization in monkeys suggests that both species might have similar neural mechanism for beat-based timing. The difference in the upper limit of predictive synchronization (1200–1800 ms in monkeys, 1800–3000 ms in humans) might come from the difference in the capacity of working memory between the species (Carruthers, 2013), or alternatively from the use of visual metronomes in the current study versus auditory metronomes in human studies on the upper limits of synchronization. Also, as discussed in more detail in Takeya et al. (2017), it remains to be seen how the brain mechanisms involved in ocular synchronization to spatialized visual metronomes are related to those involved in limb or body movement synchronization to an auditory beat, as commonly observed in humans.

## Conclusion

In conclusion, we found that monkeys trained for synchronized movements to a visual metronome could generalize predictive synchronization beyond the trained range, but also had an upper limit of synchronization, like humans. These findings suggest that monkeys may share similar synchronization mechanism with humans, which could be subject to physiological examination in future studies. If similar mechanisms are identified across these two groups of primates, it still remains to be understood why humans *spontaneously* predict when they synchronize movements with a metronome, while monkeys do not (Fuchs, 1967), and instead require extensive training to produce predictive synchronization. As suggested by the “intrinsic reward and rhythmic synchronization hypothesis” (Takeya et al., 2017), it may be that only certain vocal learning species find predictive and tempo-flexible synchronization to a beat to be intrinsically rewarding.

## Author Contributions

RT and MT conceptualized and designed the study. RT performed the experiments and analyzed the data. RT, AP, and MT wrote the manuscript.

## Conflict of Interest Statement

The authors declare that the research was conducted in the absence of any commercial or financial relationships that could be construed as a potential conflict of interest.
